# Getting to Hope: Perspectives from Patients and Caregivers Living with Chronic Childhood Illness

**DOI:** 10.3390/children8060525

**Published:** 2021-06-19

**Authors:** Emily von Scheven, Bhupinder K. Nahal, Rosa Kelekian, Christina Frenzel, Victoria Vanderpoel, Linda S. Franck

**Affiliations:** 1 Division of Pediatric Rheumatology, University of California San Francisco, San Francisco, CA 94158, USA; bhupinder.nahal@ucsf.edu (B.K.N.); rosakelekian@gmail.com (R.K.); christinafrenzel@gmail.com (C.F.); 2 Department of Hematology and Oncology, University of California San Francisco, San Francisco, CA 94158, USA; Victoria.Vanderpoel@ucsf.edu; 3 Department of Family Healthcare Nursing, University of California San Francisco, San Francisco, CA 94158, USA; Linda.Franck@ucsf.edu

**Keywords:** hope, children, chronic illness, patient engagement in research

## Abstract

Promoting hope was identified in our prior work as the top priority research question among patients and caregivers with diverse childhood-onset chronic conditions. Here, we aimed to construct a conceptual model to guide future research studies of interventions to improve hope. We conducted eight monthly virtual focus groups and one virtual workshop with patients, caregivers, and researchers to explore key constructs to inform the model. Discussions were facilitated by Patient Co-Investigators. Participants developed a definition of hope and identified promotors and inhibitors that influence the experience of hope. We utilized qualitative methods to analyze findings and organize the promotors and inhibitors of hope within three strata of the socio-ecologic framework: structural, interpersonal, and intrapersonal. Participants identified three types of interventions to promote hope: resources, navigation, and activities to promote social connection. The hope conceptual model can be used to inform the selection of interventions to assess in future research studies aimed at improving hope and the specification of outcome measures to include in hope research studies. Inclusion of the health care system in the model provides direction for identifying strategies for improving the system and places responsibility on the system to do better to promote hope among young patients with chronic illness and their caregivers.

## 1. Introduction

As advances in healthcare have led to higher survival rates for children with previously fatal conditions, the number of children and adolescents living with chronic conditions in the United States, such as asthma, obesity, mental health conditions, and neurodevelopmental disorders, has increased to over 5 million [1]. Additionally, many youths with previously fatal conditions such as certain cancers or congenital heart conditions face ongoing health challenges related to the chronicity of their disease into adulthood. These youths may develop additional health concerns and complications resulting from initial life-saving treatments, such as exposure to medications or surgical complications, which can impact their health and overall well-being [2]. Although childhood chronic illness research is an expanding field, there remain many unanswered questions about the health and social impacts of growing up with chronic illness. Additionally, there is a paucity of evidence regarding interventions to improve outcomes, particularly outcomes related to overall well-being in the context of chronic illness.

Previously, we conducted formative research to explore some of the unanswered questions posed by pediatric chronic illness patients and their families [3]. Our primary goal was to develop a research agenda that would address questions deemed important by families impacted by chronic illness. Our research priority setting working with patients and caregivers experiencing a range of chronic childhood conditions identified over 300 research questions and the following high priority topics: (1) Health Care System and Care Coordination and Communication; (2) Insurance and Health Care Coverage; (3) Patient-Parent-Provider Relationship and Communication; (4) Social, Emotional, and Family Impact and Support; and (5) Transition to Independence: Going from Pediatric to Adult care [3]. We subsequently convened a group of patients and caregivers to further select a research question with the goal of ultimately co-designing research studies to address key knowledge gaps on a high priority topic. The group reached consensus on the research question: “What can be done to promote hope? Keep aspiration for the future? Be a person outside the disease?” There was strong enthusiasm for collaborating on the topic of promoting hope and for raising awareness among clinicians and researchers about the value of promoting hope for patients and families with chronic illness.

A review of the literature revealed that hope is a universal multidimensional human construct that directly impacts quality of life and health outcomes, particularly for individuals living with chronic disease [4]. Hope is characterized by the will and determination to reach one’s goals, and touches on several related elements including personal attributes, cognitive strategies, energy, ambition, and feeling of self-worth. Numerous definitions have been proposed regarding the attributes of hope [5,6,7]. Less well studied are the promoting and inhibiting factors that influence hope. Few studies have examined hope in the context of pediatric chronic illness [8,9,10,11]. Therefore, we determined that further formative work was needed in partnership with patients and caregivers to develop a conceptual model to guide our research. Our goal was to better understand the construct of hope through the patient’s perspective so that effective interventions to improve hope can be developed. Here, we describe a co-designed conceptual model to guide future research studies that evaluate hope interventions and ultimately inform healthcare services and policy.

## 2. Materials and Methods

### 2.1. Study Design

This user-centered formative research was conducted through a series of online focus groups and two workshops with patient and caregiver partners from the UCSF Child and Adolescent Chronic Illness Center (CIC). Activities included in-person and virtual activities.

### 2.2. Participant Recruitment

Participants were recruited from our mailing list and directory of prior CIC participants, and by flier and website advertisements, at two local children’s hospitals, Benioff Children’s Hospital San Francisco (BCH-SF) and Oakland (BCH-Oak), and from two local community organizations, San Francisco Support for Families of Children with Disabilities (SFCD) and California Children’s Services, which provide a range of support services for families of children with chronic illness. The inclusion criteria for patients were: English literate and living with a chronic illness for >1 year that is anticipated to last into adulthood [12]. Examples of conditions expected to last into adulthood include sickle cell disease, epilepsy, lupus, and cystic fibrosis. Participants received a $60 Target gift card for participation in each workshop, and a $30 Target gift card for participation in each focus group.

### 2.3. Procedures

Following an initial 4-hour in-person workshop in November 2019, we held eight virtual 1-hour discussion-based focus groups (Zoom.com, accessed on 18 January 2020, 22 February 2020, 14 March 2020, 11 April 2020, 9 May 2020, 13 June 2020, 11 July 2020, and 15 August 2020, San Jose, CA, USA). The focus groups took place between January and August 2020 and aimed to gather additional information about what hope means from the patient and caregiver’s perspective. Focus group discussions were facilitated by two Patient Co-Investigators (RK, CF). Based on workshop participant feedback, sessions were held virtually in order to lower barriers to participation for participants by eliminating travel time and financial cost for the patients and families. To promote connection and community among participants and researchers, focus groups began with brief introductions and check-in activities, all related to the topic of hope (ex: what is something you do that brings you joy or makes you feel hopeful?). Participants were encouraged to keep the video on whenever possible and to contribute however they felt most comfortable (via audio or in the chat box). The poll feature in Zoom was used to conduct virtual voting during the focus groups, and an online survey was disseminated by email between focus group #4 and #5.

A final 3-hour virtual workshop was held in September 2020 to member-check our findings and seek additional insights from a larger group of patients and caregivers. Zoom breakout rooms were used to facilitate smaller group discussions. Voting was performed with virtual sticky dots on an online whiteboard (Miro.com, accessed on 12 September 2020, San Francisco, CA, USA). Participants were asked to review the previously developed list of factors that improve hope, factors that interfere with hope, and activities people do to help themselves improve hope. Through facilitated brainstorming, participants contributed additional ideas. Each participant was provided three virtual sticky dots and instructed to vote for the top three things that they personally felt were most important in the categories of: “things that promote hope” and “things that interfere with hope.”

### 2.4. Analysis

The core research team, which included 3 patient research partners, collated the material from the focus groups and workshops, and iteratively coded, categorized, and thematically analyzed the characteristics defining main attributes of hope, as well as promoters and inhibitors of hope. In keeping with the divergent (research, synthesis) and convergent (ideate, prototype) phases of a user-centered design approach [13,14], early themes were shared back to participants at subsequent sessions while continuing to iteratively explore hope attributes, promoters, and inhibitors from as many different perspectives as possible. The key domains were then organized within a socio-ecologic model according to structural, interpersonal, and intrapersonal levels of influence [15]. Concepts were then further categorized as promoters and inhibitors of hope within domains for each level. The prototype representations of hope and the model of hope influences were presented to the CIC Research Committee and in one final session with patients and caregivers to obtain any last feedback, which was then incorporated into the final model.

## 3. Results

A total of 14 patients and 25 caregivers participated in the project, with many participating in more than one activity. There were 4 patients and 11 caregivers who attended the first in-person workshop, and 10 patients and 19 caregivers who attended the final virtual workshop. Despite the co-occurrence of a global pandemic, participation remained high throughout the project. There were between 6 and 14 participants who attended each online focus group. Among the 27 participants who contributed their demographic information at the final virtual workshop, 89% were female and 11% were males with an age range of 16–24 years for patients and 35–64 years for caregivers. The ethnicity of participants was diverse (28% Asian, 9% Black/African American, 19% Hispanic/Latino, 38% White, 3% Native American/American Indian, 3% other). The chronic illnesses of these patients and the children of the caregivers included ulcerative colitis, post-traumatic stress disorder, depression, Crohn’s disease, lupus nephritis, epilepsy, sickle cell disease, and Blau syndrome, among other chronic conditions.

The discussion prompts from the monthly online focus groups are summarized in Table 1.

### 3.1. The Idea of Hope

**Figure 1:**
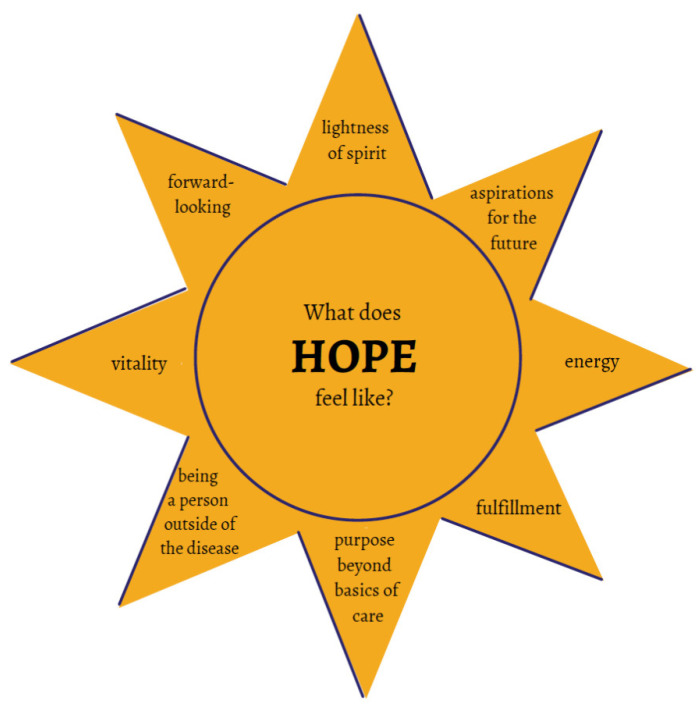
What does HOPE feel like? This patient and family-generated graphic depicts what hope feels like to them. This conceptualization of the attributes of hope can be used to guide the selection of outcome measures for future research studies aimed at improving hope.

Participants described what hope meant to them (Figure 1). They described how the term “hope” was associated with the experience of having aspirations and purpose because this allowed them to feel like they were “a person outside of the disease” and provided something to look forward to. They identified the feeling of vitality and lightness of spirit, which they felt allowed for energy to apply towards their goals and a way of being that allowed for them to “unbury” interests and passions.

### 3.2. Promoters and Inhibitors of Hope

**Figure 2:**
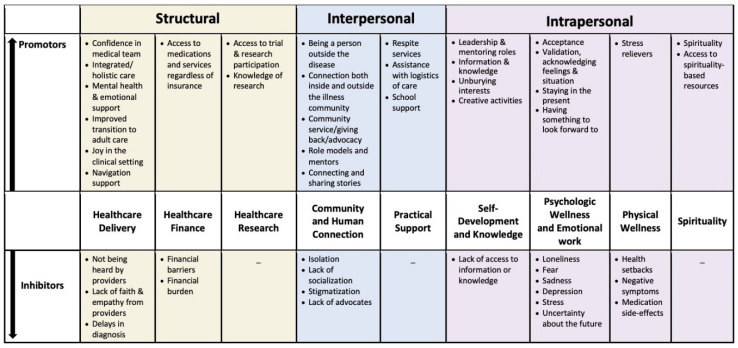
Summary of hope promotors and inhibitors. The comprehensive list of factors that influence hope derived from the participants were organized by promoters, inhibitors, and level of influence. The items selected by the participants as being of greatest importance are presented here.

Within the three levels of socioecological influence (i.e., structural, interpersonal, and intrapersonal), patients and caregivers described numerous factors that either promoted or inhibited hope (Figure 2 and Table S1). The number of factors of greatest importance varied within and across level of influence and domain. The largest number of examples were provided by participants for the domains of healthcare delivery, community and human connection, and psychological and emotional well-being. The patients and caregivers did not provide specific examples of inhibitors within the domains of healthcare research, practical support, or spirituality. 

Important promotors of hope within the healthcare delivery domain included having confidence in the medical team and joy in the clinical setting. In contrast, poor communication with providers and lack of empathy from providers were described as inhibitors of hope.

Participants identified community as an important promotor of hope through improving connection, providing opportunities for role models, and allowing for “stepping outside of yourself.” They described two main types of community, both of which had an important influence on hope: community of people with chronic illness, and community of people without chronic illness. They pointed to uncertainty and use of language as potential barriers to hope. An example of language that hindered hope was the use of “we worry that….” by providers.

Participants identified the following three types of interventions to promote hope: resources, navigation, and social connection. In addition, participants generated several ideas for specific interventions to be further evaluated (Table 2). They recommended the following types of resources that could be housed in an online hub: podcasts, videos, chat rooms, or a living document template to keep track of health information. They suggested that the process of resource navigation would require support by a person who could form an emotional connection with a patient, engage in an “interview” to identify needs, and connect patients with resources. Participants identified two approaches to fostering social connection: peer programs and “action-oriented” groups such as volunteer activities. They acknowledged that sometimes it is difficult for people to take advantage of social support activities, and emphasized the importance of ensuring a “hook” to engage people. Participants expressed views that in-person gatherings were generally more effective at developing relationships between participants, but once formed, the group could transition to virtual platforms.

Through an online survey between focus group #4 and #5, participants were asked to rank the following types of interventions from most to least important to study through research: activities that bring chronic illness patients together socially such as support group activities, camp, or events; activities that teach emotional wellness such as psychotherapy, mindfulness-based treatments, cognitive behavioral therapies, or journaling; activities that give 1-on-1 help such as a personal coach, buddy, peer counselor, or individual peer support; activities that give patients a “purpose” beyond chronic illness such as involving role-models or activities that promote purpose beyond managing one’s illness; and activities that give patients ways to get involved in kind acts or service to others such as volunteering activities to help others. The participants ranked activities that bring chronic illness patients together socially as most important. When participants were asked which was of higher priority,—developing an intervention that targeted the patient, the parent/caregiver, the siblings, the whole family, friends, or the healthcare provider,—the participants ranked the patient as most important to receive the intervention.

**Figure 3:**
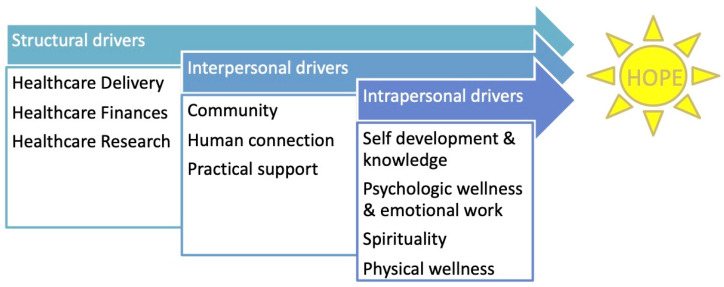
Conceptual Model: Multiple Pathways to Hope. Analysis revealed three parallel pathways to hope, based on structural, interpersonal, and intrapersonal levels of influence.

Figure 3 depicts the final conceptual model of hope, with three main pathways to hope, based on structural, interpersonal, and intrapersonal levels of influence. The structural level included domains related to healthcare delivery, healthcare finance, and healthcare research. The interpersonal level included domains related to community and human connection and practical support, and the intrapersonal level included domains related to self-development and knowledge, psychological wellness and emotional work, physical wellness, and spirituality.

## 4. Discussion

Our patient-and-family partnered approach to research prioritization, which utilized the Research Prioritization by Affected Communities (RPAC) method [16], led to the specific research focus of promoting hope as a potential intervention for improving quality of care and quality of life for children with chronic illness [3]. However, we discovered that further formative work was needed to fully conceptualize hope within the context of pediatric chronic illness before embarking on designing research studies of hope-promoting interventions. Using a user-centered design approach, patients, caregivers, researchers and clinicians co-created a new conceptual model of hope that includes a holistic definition of hope and three parallel pathways to hope with multiple promotors and inhibitors, based on the lived experience of patients with pediatric-onset chronic illness and their caregivers.

### 4.1. Defining Hope

Prior conceptualizations of hope narrowly defined it in relation to therapeutic goals and future aspirations, and emphasized intrapersonal characteristics [17]. A thematic analysis of qualitative studies addressing the experience of hope among children with chronic illness by Leite et al. identified five themes: uncertainty, support, information, between “dark thoughts” and positive thoughts, and hoping to go back to normality [11]. The attributes of hope identified in our study (Figure 1) included internal states of vitality and energy, future focus, as well as characterization of personal identity and one’s value and potential for impact on the world. Moreover, the future focused concepts arising from our work were more focused around hoping to get a new and better future that encompassed the chronic illness, in contrast to previous definitions that focused on “hoping to go back to normalcy” [11], which implies that there could be no hope without the reversal of the disease. It was clear in all of our interactions with patients and caregivers that a definition of hope that centered on a prior “normal” state or a narrowly defined goal, was inconsistent with their lived experiences, and would be unlikely to respond to interventions in a clinical trial aimed at improving hope, or ultimately improve well-being.

### 4.2. Conceptual Models of Hope

Several theories and frameworks of hope have previously been proposed. The Hope Theory, developed by Snyder, incorporates three elements: goals, pathways thinking, and agency [18]. Pathways thinking refers to the ability to identify many different routes to a goal. Agency refers to the ability to initiate and sustain motivation to reach a goal. The Hope Intervention Model proposed by Farran and Popovich describes intervention domains to promote hope in geriatric patients, including: stressful life events, physical health, mental health, social support, interpersonal control, and interactive and global hope [19]. These models were developed from adult populations with data from a predominance of acutely ill or palliative care patients. Shaw et al., in a systematic review of interventions aimed at improving mental health and well-being among children with chronic illness, proposed a model that outlines bidirectional relationships between five key constructs: Getting in and Staying in, Therapeutic Foundation, Social Support, a Hopeful Alternative, and Empowerment [20]. Getting in and Staying in addresses issues of availability, accessibility, engagement, and keeping it going, and provides some guidance around the characteristics of a successful intervention.

In contrast, our work with young people with childhood-onset chronic illness and their caregivers has generated a model with three separate pathways, distinguished by the level of socioecological influence: structural, interpersonal, and intrapersonal. The possibility that hope can be promoted (or inhibited) along any of the pathways simultaneously provides a wider range of options for intervention and the parallel pathways may provide redundancy, which offers more possibility for achieving hope, as additional emphasis can be given to one pathway if another is blocked. This hypothesis about redundancy should be addressed in future research. A multiple pathway model is consistent with strength-based positive psychology approaches that focus on the positive attributes rather than the negative ones and do not require eradication of problematic features to reach success [21,22].

Resilience, a psychologic construct that refers to either one’s ability to cope with adversity [23,24] or the process of successfully adapting to stressful situations, is an important asset in the context of chronic illness. Hope has been described as a driver of resiliency [25]. However, the relationship between hope and resiliency may be more appropriately characterized as a bidirectional relationship, wherein resiliency in an adverse situation may lead to greater hopefulness, as well as hopefulness leading to greater resiliency. Positive childhood experiences have been shown to associate with mental health outcomes [26] and may also be promotors of hope. The Healthy Outcomes from Positive Experiences (HOPE) framework [27] was created to address the contribution of positive childhood experiences to improved resiliency and health outcomes. Recognition of the contribution of positive childhood experiences provides an approach for examining modifiers of adverse childhood experiences, and establishes a language for developing strategies to enhance positive experiences. Some of the promotors and inhibitors of hope suggested by participants in this project may be characteristics of positive childhood experiences and adverse childhood experiences, respectively. However, we report drivers of hope expressed by patients and caregivers who are living with chronic childhood illness, rather than using the taxonomy of the research or mental health community.

The situating of our hope conceptual model within a socioecological framework is in contrast with the previous more intrapersonal and psychologic-based models of hope [18]. This allows a more holistic assessment of drivers of hope and provides greater opportunity for a range of interventions to be explored. A broader definition of the multiple attributes of hope and the pathways of influence affecting the person (intrapersonal) in the context of their social connections (interpersonal) and the entire healthcare system (structural) is well suited for examination of hope for children living with chronic illness and their families. Additionally, the inclusion of the health care system in our model lays the foundation for identifying strategies for improving the system and places responsibility on the system to do better to promote hope among young patients with chronic illness and their caregivers.

The role of social support, emphasized in the model by Shaw et al. [20], is also reflected in the interpersonal domain of our proposed model. However, in contrast with our conceptual model, Shaw’s model does not include the larger role of the healthcare system as a driver of hope, and focuses on the outcomes of hope through the pathologic lens of mental health. The patients and caregivers who participated in our project told us clearly and repeatedly how strongly the health system and healthcare providers both positively and negatively influence their degree of hopefulness. This difference between our model and Shaw’s may reflect that unlike the pediatric model derived from a literature review by Shaw et al., our model was co-developed with patients and caregivers. We received a clear call to action to conduct intervention studies aimed at improving hope-promoting interventions within healthcare delivery.

### 4.3. Hope Research

Our main goal with this project was to develop a conceptual model to guide future research studies, including the selection of interventions and the specification of outcomes and other important factors that should be measured. Proposed interventions arising from our formative work include more mental health support, coaching, community building activities, easy and immediate access to a medical professional for questions, and “fun” things at the hospital. The participants in this project made it clear that the desired interventions were not related to their underlying conditions but rather to the individual’s personal needs and circumstances. Similarly, there was no consensus on the optimal format for interventions, with online, digital, and in-person modalities all felt to be valuable. Interestingly, patients indicated that virtual engagement for peer support is more effective if there is an in-person meeting first to facilitate establishing connection and relationship. These findings challenge researchers to utilize effectiveness-implementation hybrid study designs that allow for both the evaluation of personalized interventions and implementation strategies [28]. Even though more complex, such research may yield more effective and actionable findings that will more readily be implemented in practice.

### 4.4. Hope in Healthcare

Although our primary aim was to develop a conceptual model to guide patient-and family partnered research to improve well-being in pediatric chronic illness, our work yielded findings that can be implemented in practice immediately without further research. For example, there is already sufficient evidence demonstrating the importance of patient-provider communication, trust, and shared decision-making for improving health outcomes [29,30]. However, we learned from our participants that clinical practice is not always consistent with this evidence. Similarly, despite what is known about the importance of psychological and social support in chronic illness, it was clear in our work that these resources are not consistently provided to patients. Ensuring that all patients and families have access to basic support services and that healthcare teams practice patient-and family-centered care should provide the foundation on which to build future interventions to promote hope and further improve outcomes over the life course in patients with childhood chronic illness.

### 4.5. Limitations

The novel conceptual model developed from this formative work will require further research with multiple diverse cohorts to determine its generalizability and utility in guiding hope research and interventions across a broader range of pediatric chronic illness patients and caregiver populations, including those who speak other languages. Specific areas to be explored in future research with the model include identification of exemplars of inhibitors of hope within the domains of healthcare research, practical support, or spirituality. Additionally, despite the diversity of the patient participants, culturally specific promotors and inhibitors of hope were not identified and, thus, require further research. Further user-centered design studies could begin with this initial model and solicit direct feedback from the participants to identify factors within each domain, reflecting on their own lived experience. Finally, the model will require empirical testing to determine its utility to explain or predict hypothesized relationships or model-based interventions.

## 5. Conclusions

In summary, our findings add to the literature on hope among patients living with pediatric-onset chronic illness and their caregivers by anchoring the factors that drive hope, situated within a socio-ecologic framework. This extends the focus of our understanding of hope in chronic illness beyond the individual’s emotional and psychologic state to include their entire social and societal environment, including the healthcare system, which is a dominate presence in their daily lives. Our work was uniquely conducted in full partnership with patients and caregivers, adding the authenticity of lived-experience and the rigor of co-design to the process of conceptual model development.

In addition to deepening our understanding of hope through the lens of patients living with chronic childhood illness and their caregivers, we aimed to construct a conceptual model to inform future research studies. We have identified several areas to target interventions, across three levels of influence: structural, interpersonal, and intrapersonal. This model informs the selection of interventions to be assessed in future research, in addition to the specification of outcome measures to include in hope research studies. Future research aimed at improving our understanding of the influencing factors and identifying ways to deliver interventions through a personalized medicine approach should help improve hope for the increasing number of children who are growing up with chronic illness.

## Figures and Tables

**Table 1:** Description of discussion prompts used at each virtual focus group to explore the concept of hope and characteristics of interventions that promote hope among patients and caregivers.

Activity	Discussion Prompts
Focus group #1	What does hope mean to you, as a patient/caregiver? What might hope “look like” for chronic illness patients? What factors do you think contribute to, or deter from hope?
Focus group #2	How might we measure hope? How do we know if hope has been achieved or improved? What does it feel like? What do you do when you have hope? In contrast, in the absence of hope, what happens?
Focus group #3	What types of interventions might help improve hope for patients/families?
Focus group #4	What should our intervention focus on? Who should be involved in our intervention? Patients, caregivers, siblings, whole family, community, or providers? How should interventions be delivered? Web-based, app-based, therapy, peer support, education, community-building, other?
Focus group #5	Thinking about past social support: ○ What’s worked for you/your child, and why? ○ What hasn’t been helpful, and why? ○ What kind of social resources do you wish you’d had in the past, or would be helpful in the future?
Focus group #6	What factors influence the kinds of support that work best for you? What settings feel most safe or helpful for you when receiving support? How might patients/families be matched with support that best fits their needs? What aspects of social and emotional support feel most important to you?
Focus group #7	How can we best match patients/families to the support programs they need? What are some ways that would be useful to help you find support? How do we make support programs accessible? How do we make support more enticing so patients want to be involved, and don’t feel embarrassed, shameful or stigmatized? How can we ensure that support programs address our initial goal of improving hope for chronic illness patients?
Focus group #8	What type of support would you like us to research in a future study?

Key components of the final hope conceptual model are described below.

**Table 2:** Specific interventions to promote hope. Patients and caregivers developed the list through structured brainstorm and discussion over a series of eight focus groups.

Social connection (e.g., buddy (friend with similar chronic illness), mentor (someone with more experience or older living with chronic illness), support group, chat rooms, online communities) Advocacy and volunteer activities Online resources (e.g., podcasts, videos, menu of options for engagement) Someone or something fun to look forward to at the hospital (e.g., therapy dog or an art activity) An empathetic physician who checks in regularly A medical professional “I can speak to right away” A point person to help (e.g., navigator or help-desk) Psychologist Art therapy School counselor Religious community Everything should include a “hook” to get people involved

## Data Availability

The data presented in this study are available in Table S1: Promotors and inhibitors of hope generated by patients and caregivers.

## Supplementary Materials

The following are available online at https://www.mdpi.com/article/10.3390/children8060525/s1, Table S1: Promotors and inhibitors of hope generated by patients and caregivers.
