# Supplementary material for: Getting to Hope: Perspectives from Patients and Caregivers Living with Chronic Childhood Illness
Source: Children (Basel). 2021 Jun 19;8(6):525. doi: 10.3390/children8060525 (PMC8235585; doi:10.3390/children8060525)
Supplement: Supplementary file 1 [file children-08-00525-s001.zip › children-1223317-supplementary.pdf]

children

Supplementary Materials

Table S1. Promotors and inhibitors of hope generated by patients and caregivers.

STRUCTURAL LEVEL<br>Domain	Promotors	Inhibitors
Healthcare research	• Access to research, knowledge/awareness of research for improving healthcare<br>• Knowledge/awareness/pursuit of research going on to improve the healthcare system<br>• Access to clinical trials<br>• Research related to illness	
Healthcare finance	• Access to all medications and services, regardless of insurance<br>• Medical care for all; no insurance issues	• Financial barriers; financial burden of ongoing medical bills*
Healthcare delivery	• Confidence in medical team that supports us*<br>• More integrated, holistic, alternative medical approaches*<br>• More mental health and emotional support*<br>• Better transition from child to adult care*<br>• Second opinions or options beyond medical care<br>• Transition care<br>• More support/follow up at home<br>• Easier access/connection with providers (accessibility)<br>• support<br>• Trust based patient/doctor relationship<br>• More meaningful communication between medical teams<br>• Access to social workers<br>• Simplified questionnaires/assessments<br>• More patient involvement in care<br>• more involvement from patient with doctors/parents<br>• Joy within the clinical setting (therapy animals, role model visits)<br>• navigating healthcare system/patient navigator<br>• better laws/policies to support communication between patients and doctors	• Not being heard by providers; lack of faith, empathy, sensitivity, compassion, and attitude from doctors; being dismissed (provider)*<br>• Difficulty managing logistics (insurance, appointments, prescriptions)<br>• Feeling your care is lacking, not giving you everything you need (e.g., Struggling to get a port)<br>• When others don't listen to you/your needs (provider)<br>• Words people use (provider)<br>• Lack of sensitivity, compassion, mindfulness, around privacy (provider)<br>• Lack of communication between providers<br>• negative/neutral delivery of news from doctors (provider)<br>• Delays in diagnosis<br>• Broken health system<br>• Language and cultural barriers for access to service
**INTERPERSONAL LEVEL**		
Community & human connection	• Being a person outside the disease*<br>• Community service (giving back)<br>• Community/human connection outside illness community<br>• Community/connection inside chronic illness community<br>• Get support from others (peer support)<br>• Sharing stories, hearing positive patient stories<br>• Connecting with friends (in/out of hospital)<br>• Helping family around the house<br>• Getting involved (advocacy)<br>• Getting involved in politics<br>• Enjoyable activities (retail therapy, being outdoors, games, food/wine tours, pets, nails/hair done)<br>• Camps related to illness<br>• Mentorship programs; having a mentor<br>• Positive patient stories<br>• Hope role models<br>• Family based activities	• Not having connection to advocates<br>• Isolation<br>• Lack of socialization<br>• Stigmatization
Practical support	• Having resources that give us more time to do 'stress-relieving' activities	

	• Get help with logistics of care to allow time for enjoyable activities<br>• Respite services (e.g., various home help- errands, cleaning etc.)<br>• Help from schools (support services)	
**INTRAPERSONAL LEVEL**		
Spirituality	• Spirituality and access to spirituality-based resources<br>• Faith/ Daily prayers	
Self-development & Knowledge	• Taking a leadership or mentoring role yourself<br>• Identifying goals and specific aspirations<br>• Aspirations for the future<br>• Get more information (consult resources)<br>• Knowledge<br>• Self-care<br>• Unburying interests<br>• Art, music & other creative activities<br>• Traveling<br>• Planning for the future (e.g., job, travel)	• Lack of access to information or knowledge
Psychologic wellness & emotional work	• Acceptance<br>• Acceptance of new normal<br>• Validation/acknowledgement of feelings and situation<br>• Willingness to be vulnerable<br>• Getting support from a professional (e.g., therapist, SW, etc.)<br>• Having a routine<br>• Staying in the present moment/day<br>• Bringing things to look forward to into hospital setting<br>• Something to look forward to<br>• Not aiming for perfection (e.g., accepting child as they are)	• Uncertainty about the future*<br>• Feeling alone, fear, sadness, depression, and stress*
Physical wellness	• Stress relievers (e.g., movement, anything that gets me in motion)	• Health setbacks<br>• Harmful impacts of medications<br>• Pain and other physical impacts

Items are separated according to level of influence (structural, interpersonal, intrapersonal) and domain. * indicated promotors and inhibitors that were rated highly (≥6 votes) for importance by patients and caregivers .
